# Negative interpretation of ambiguous bodily symptoms among illness-anxious individuals: Exploring the role of developmental and maintenance constructs

**DOI:** 10.3389/fpsyt.2022.985125

**Published:** 2023-01-09

**Authors:** Mina Elhamiasl, Mohsen Dehghani, Mahmood Heidari, Linda M. G. Vancleef, Ali Khatibi

**Affiliations:** ^1^Psychology Department, Shahid Beheshti University, Tehran, Iran; ^2^Section Experimental Health Psychology, Department of Clinical Psychological Science, Maastricht University, Maastricht, Netherlands; ^3^Centre of Precision Rehabilitation for Spinal Pain, School of Sport, Exercise and Rehabilitation Sciences, University of Birmingham, Birmingham, United Kingdom; ^4^Centre for Human Brain Health, University of Birmingham, Birmingham, United Kingdom; ^5^Institute for Mental Health, University of Birmingham, Birmingham, United Kingdom

**Keywords:** illness anxiety, interpretation bias, reappraisal, intolerance of uncertainty, anxiety sensitivity, bodily symptoms, COVID-19

## Abstract

**Background:**

Cognitive factors play an essential role in the development and maintenance of anxiety problems. Among individuals with illness anxiety problems, their interpretation of bodily symptoms is a crucial factor in the determination of their ability to regulate their emotions. The catastrophic interpretation of ambiguous bodily symptoms and changes, known as interpretation bias, in line with the failure to reappraise the symptoms in safer ways, is supposed to increase the levels of anxiety in illness-anxious individuals.

**Methods:**

This study aimed to address the statistical limitations of the direct (self-report) measure of interpretation bias, using an indirect (online interpretation bias task) measure for assessing biased interpretations of bodily symptoms. In addition, we examined the contribution of self-report anxiety sensitivity (AS), intolerance of uncertainty (IU), interpretation bias, and reappraisal to illness anxiety problems in a subclinical population and compared it with controls with low levels of illness anxiety.

**Findings:**

Illness-anxious individuals made more negative interpretations of ambiguous, potentially health-threatening information. They used less reappraisal to regulate their emotion. Among the measures, the physical subscale of AS and the reaction time to the safe resolution of ambiguous information were the best factors that could contribute to the differentiation between the illness-anxious individuals and non-anxious individuals.

**Conclusion:**

Our findings provided further support for the biased processing of information related to physical symptoms among individuals with illness anxiety. AS-physical and safe resolutions for ambiguous situations could differentiate the illness-anxious and the control groups better than other factors. These findings suggest that a change of interpretation of ambiguous bodily symptoms among individuals suffering from chronic conditions can be a possible intervention to target anxiety and improve patients' lives.

## Introduction

Illness anxiety disorder, formerly known as hypochondria, is characterized as dysfunctional worry about serious illnesses (1). In addition, illness anxiety can be experienced as a symptom in people suffering from other disorders, such as GAD, specific phobia, and OCD (2, 3). The experience of illness anxiety has increased since the emergence of the COVID-19 (SARS-CoV-2) pandemic in December 2019, as it has caused notable physical and psychological distress as well as high mortality and morbidity rates (4). Studies showed that during the pandemic, people had concerns about COVID-19 and a considerable portion of them reported that their level of anxiety and psychological stress increased during the pandemic (5). Anxiety and fear of COVID-19 were also reported to worsen preexisting mental health problems and lead to post-traumatic stress disorder (PTSD) and even suicidal thoughts and attempts (6–8).

Some studies propose a developmental perspective to explain susceptibility or resilience to mental problems (9). Furthermore, studies suggest that long-term exposure to stress can result in changes in physiological functioning, which can lead to long-lasting effects and the development of long-term consequences (10, 11). According to the cognitive behavioral approach to illness anxiety (12), the core feature of illness anxiety is the catastrophic interpretation of ambiguous bodily symptoms and changes, known as interpretation bias. Biased interpretations may increase hypervigilance of the internal sensations (i.e., body sensations) (13–15). These perceived sensations, in turn, can be interpreted as signs of a serious disease and consequently increase the primary dysfunctional catastrophic interpretations (12, 16). These catastrophic interpretations, then, might be maintained as individuals may make less effortful reappraisal strategies to replace these negative interpretations with safer ones. Thus, they might experience more dysfunctional anxiety in response to the maintained negative interpretations. Reappraisal is one of the emotion regulation strategies that refer to altering the meaning of a stimulus or a situation to reduce negative emotions (17, 18). A negative relationship between reappraisal strategy and interpretation bias for health-threatening information has been established by several studies (19–21). Therefore, the biased interpretation of health-related information and deficit in further reappraisal regulatory strategy might be two maintaining factors contributing to the maintenance of illness anxiety disorder.

Anxiety sensitivity (AS) and intolerance of uncertainty (IU) are suggested as two psychological constructs that may make people vulnerable to illness anxiety disorder and the experience of interpretation bias when facing physical sensations. AS is defined as the experience of fear or worry over the symptoms that are associated with anxiety (22). Sensitivity to arousal-related sensations makes individuals misinterpret harmless bodily sensations and symptoms of a medical problem, which may lead to illness anxiety (23). The experienced anxiety, in turn, might increase the severity of perceived anxiety-related symptoms convincing the individual that a health problem exists. The somatosensory amplification model of hypochondriasis supports the role of AS in illness anxiety, stating that people with illness anxiety are more sensitive to normal sensations and interpret them as signs of a serious disease (24).

A low threshold for tolerating the unknown nature and consequences of bodily signs may contribute to IU. People with IU believe that uncertainty and ambiguity are negative and attempt to put an end to this ambiguity by considering the most catastrophic consequences (25). As such, individuals with illness anxiety may consider ambiguous bodily sensations as indicators of a serious medical disease since they are less tolerant of ambiguity (23).

Despite the growing body of evidence about the relationship between catastrophic interpretations and negative emotions such as anxiety, few studies have compared the relationship between anxiety and interpretation bias among people with and without illness anxiety symptoms. Such a comparison can highlight the role of negative interpretation bias in illness anxiety. In addition, the studies about illness anxiety and catastrophic interpretations mostly relied on direct self-report measures of interpretation bias. These measurement methods are reported to be subject to respondent bias (13). Therefore, indirect measures would be more appropriate for assessing these biased interpretations.

It is important to consider that illness anxiety is a common disorder, with estimates ranging up to 13% in the general adult population (26). Furthermore, the emergence of new diseases (for example, COVID-19 and monkeypox) in recent years and their uncertain health outcomes have triggered higher levels of illness anxiety. With regard to the prevalence of illness anxiety and the importance of comprehending the cognitive factors that are involved in its development and maintenance, the current study was designed to examine the role of cognitive factors in the development and maintenance of illness anxiety by comparing individuals with and without illness anxiety symptoms. Unlike previous studies, we aimed to measure interpretation bias for ambiguous bodily symptoms using an indirect online interpretation bias task (27). We believe that the application of this novel method would assist us in understanding the role of biased interpretations in differences among people with and without illness anxiety symptoms. We hypothesized that individuals with illness anxiety symptoms show more catastrophic interpretations of ambiguous health-related situations. We also investigated the levels of reappraisal, AS, and IU in individuals with illness anxiety symptoms and compared them with those without these symptoms. We hypothesized that the illness-anxious participants would report higher levels of AS, IU, and interpretation bias but less reappraisal in comparison to the individuals without illness anxiety symptoms. More importantly, we aimed to examine which factor among interpretation bias, appraisal, AS, and IU had the most power in differentiating the illness-anxious group from the control group.

## Method

### Participants

A power analysis, based on previously published and unpublished pilot studies, investigated the interpretation bias among individuals with high and low levels of illness anxiety (28) and suggested 29 participants in each group to explore the between-subject differences. Participants were 60 students of Shahid Beheshti University [two groups: illness-anxious and control (30 in each)]. First, the participants for the illness-anxious group were recruited *via* ads in public places of the university that called for individuals with illness anxiety symptoms specified in the ad. A semi-structured interview was used to confirm the presence of illness anxiety symptoms. Then, the matched participants for the control group were recruited using an announcement inviting individuals without illness anxiety symptoms.

#### Illness-anxious group

The illness-anxious sample included 30 (15 female) students of Shahid Beheshti University selected through an announcement calling for individuals with illness anxiety symptoms. In this announcement, illness anxiety symptoms, including experiencing worries about health, checking body status, avoidance of health-related information or searching for health-related information, and being sensitive to bodily changes, were listed. Volunteer students were supposed to inform the experimenter using email or SMS. To clarify the presence of illness anxiety symptoms, a semi-structured interview assessing illness anxiety symptoms was conducted by one of the authors (M.E.) who holds a master's degree in clinical psychology. Participants who are suffering from a serious medical condition, are under medical or psychiatric medication, have a history of surgery in the last 12 months, have a strong belief in already suffering from a disease that cannot be diagnosed by physicians, and suffering from psychotic disorders were excluded. Regarding the illness anxiety symptoms as well as exclusion criteria, 30 students (15 female) were selected among 47 volunteer students. Other 17 participants were excluded due to not meeting the criteria for illness anxiety symptoms (6), suffering from a medical disease (3), having a history or suffering from another psychiatric disorder (4), using psychiatric medications (2), and being under psychotherapy (2).

#### Control group

The control group consisted of 30 students at Shahid Beheshti University selected through an announcement requesting individuals without illness anxiety symptoms. Volunteer students were supposed to inform the experimenter using email or SMS. Volunteers whose gender and age range matched the illness-anxious individuals were invited to the interview session. To clarify the absence of illness anxiety symptoms, a semi-structured interview assessing illness anxiety symptoms was conducted. Suffering from a serious medical condition, being under medical or psychiatric medication, having a history of surgery during recent 12 months, and suffering from psychotic disorders were the exclusion criteria. A total of 30 students (15 female) among 38 volunteers were determined as eligible to be studied as the control group. Notably, eight volunteers were excluded due to suffering from a medical disease (2), a history of suffering from another psychiatric disorder (3), using psychiatric medications (2), and a recent history of surgery (1). However, one of the volunteers could not attend the measurement session due to an unexpected medical problem, and the sample size was reduced to 29 participants.

### Measures

#### The structured clinical interview for DSM-5, clinician version (SCID-5-CV)

The SCID-5-CV (29) is a semi-structured interview guide to make the DSM-5 diagnoses. In this study, however, it was used to check illness anxiety symptoms, including (1) preoccupation with illness in the absence of somatic symptoms, (2) experiencing anxiety regarding illness-related issues, and (3) avoidance or safety behaviors. The experience of all these three symptoms during the last 6 months was our criteria for inclusion in the illness-anxious group. It was administered by the author (M.E.) who holds a certified degree in clinical psychology. The purpose of using this tool was not to diagnose participants with IAD or determine the severity but to check the existence and absence of the aforementioned symptoms, respectively, in the illness-anxious and control groups.

#### Interpretation task

The interpretation task was specifically designed for the study and was modeled after the online interpretation paradigm as described by Vancleef et al. (27). The task assesses interpretation bias for ambiguous health-related information. It consisted of 32 scenarios with a length of four lines, of which the final sentence of each description is incomplete and lacks a word. The task incorporates 16 ambiguous (AMB) scenarios that can have both an unsafe (health-threatening) or a safe resolution. Furthermore, it includes 16 forced inference scenarios, of which 8 are health-threatening (HR) such that only the unsafe resolution makes sense for that scenario (endorsing safe resolutions are considered as errors) while the other 8 are non-health-related (NHR) such that only the safe resolutions make sense (endorsing unsafe resolutions are considered as errors). A total of eight ambiguous scenarios are matched to eight health-related scenarios (the HR resolution of the ambiguous is the same as the correct resolution of the forced), while eight ambiguous scenarios are matched to eight non-health-related scenarios (the NHR resolution of the ambiguous is the same as the correct resolution of the forced). The forced inference scenarios are used as control scenarios. Each task trial started with a fixation point presented on the screen for 500 ms. Then, the first line of a scenario was shown on the screen for 2,000 ms, and after that, the second to the fourth lines (each for 1,500 ms) were consecutively added. The fourth line contained a missing word. In the ambiguous descriptions, the missing word maintained the ambiguity of the scenario. Next, at 7,000 ms, one unsafe and one safe word were simultaneously presented on screen for a total of 3,000 ms while the scenarios were still on the screen. The participant was instructed to start reading the scenario line by line as soon as it appeared on the screen. When the two words were presented, the participant's task was to choose the word that completed the story in the way they thought. They were instructed to make this choice as soon as possible. As soon as the subject pressed the key, the next trial started with the presentation of a fixation point. Although we had a control group to control any confounding variables, such as the speed of reading the scenarios and resolutions, the readability of both resolutions, that is, their length, was similar.

Interpretation bias was indexed by valence and reaction time scores. A negative interpretation bias-valence refers to the smaller number of safe resolutions and the greater number of unsafe resolutions for ambiguous scenarios. A negative interpretation is also expected to result in faster reaction times when the subject has chosen the unsafe resolutions of ambiguous scenarios than the safe ones.

The current task was the modified and summarized version of the original Dutch version of the online interpretation task developed by Vancleef et al. (27). The mean of latency for safe resolutions (ambiguous safe and forced safe conditions) was less than the latency for health-threatening resolutions (ambiguous health-threatening and forced health-threatening conditions) (27). Lower levels of latency (i.e., faster reaction time) can reflect that the scenarios can validly differentiate health-threatening interpretations from safe ones. The scenarios were translated into Farsi and adapted based on the cultural context. The accuracy of the translation was assessed by a person holding a degree in Farsi literature as well as two clinical psychologists holding a PhD degree in clinical psychology. Then, 10 students from other majors than psychology read and informed us if the scenarios were understandable. They also evaluated if sentences were correctly categorized in each of the AMB, HR, and NHR trials. In addition, these students were asked to perform the computerized task to see if reading and comprehension of the scenarios are possible in different periods (5,000, 6,500, and 8,000 ms). A total presentation time of 6,500 ms was selected as appropriate, providing enough time to read the scenarios fully while not allowing additional time to (re)think about them. The task was developed using the Affect 4.0 program (30). The task also had a training phase consisting of 10 non-health-related scenarios that were different from the main scenarios. The training phase was performed to get participants acquainted with the response procedure. Refer to Appendix A for some examples of the scenarios translated into English.

#### Anxiety sensitivity index (ASI)

The Anxiety Sensitivity Index (31) is a 16-item questionnaire that assesses the fear of somatic and cognitive symptoms of anxiety. ASI has three subscales, including physical concerns, mental incapacitation concerns, and social concerns (32). Each item is rated on a five-point Likert scale (0 = very little; 4 = very much). The psychometric properties and predictive validity have been well-approved (32). The internal consistency of the Farsi version of this questionnaire, calculated by Cronbach's alpha, was 0.90 in the current study.

#### Intolerance of uncertainty scale (IUS)

The Intolerance of Uncertainty Scale (33) is a 27-item questionnaire that measures the inability to tolerate uncertain situations. Participants are asked to rate items on a five-point Likert scale (1 = “not at all characteristic of me” to 5 = “entirely characteristic of me”). It has two sub-factors, including uncertainty having negative behavioral and self-referent implications (factor 1; 15 items) and uncertainty being unfair and spoiling everything (factor 2; 12 items). The total scale has excellent internal consistency and good test–retest reliability (34). The internal consistency of the Farsi version of this questionnaire, calculated by Cronbach's alpha, was 0.96 in the current study.

#### Emotion regulation questionnaire (ERQ)

The Emotion Regulation Questionnaire (35) is a 10-item scale designed to measure cognitive reappraisal and expressive suppression. Participants rate their answers on a seven-point Likert Scale (1 = strongly disagree, 4 = neutral, and 7 = strongly agree). The original internal consistency of the questionnaire was reported as appropriate (35). We used the reappraisal subscale in the current study. The internal consistency of the Farsi version of the reappraisal subscale of this questionnaire, calculated by Cronbach's alpha, was 0.84 in the current study.

#### Cognitions about body and health questionnaire (CABAH)

The Cognitions about Body and Health Questionnaire (36) is a 31-item questionnaire assessing five subscales of catastrophizing interpretation of bodily complaints, autonomic sensations, bodily weakness, intolerance of bodily complaints, and health habits. Items are rated on a four-point Likert Scale (0 = completely wrong, 3 = completely right). The internal consistencies of this questionnaire in the clinical and normal samples were reported as 0.90 and 0.80, respectively (36). The internal consistency of the Farsi version of this questionnaire, calculated by Cronbach's alpha, was 0.93 in the current study. In the current study, we only used the catastrophizing interpretation of the bodily complaints subscale (CABAH-Cat) as the index of self-report (and direct measure of) interpretation bias toward health-related information.

#### Procedure

The study was approved by the Ethical Committee of the Department of Psychology at Shahid Beheshti University (Ref Number: 30514). The ads for recruiting the illness-anxious participants were put in public places in the university. Upon the expression of interest and our favored gender ratio (15 female and 15 male), volunteers were individually invited to an interview session held at the laboratory in the Department of Psychology. Participants who met inclusion criteria but not exclusion criteria were informed by the interviewer (M.E.) that they were selected for further examinations. The interviewer also informed the participants briefly that the current study consisted of completing questionnaires and one computerized task. Then, participants were asked to read the consent form describing research aims and procedures, as well as possible advantages and risks of the study, and sign it in case they agreed with the provided information. After obtaining informed consent, the participant and experimenter (M.E.) scheduled a date for the test appointment.

The test appointment session started in the psychology laboratory where participants completed the questionnaires. After completing the questionnaire battery, participants were taken to the test laboratory equipped with desktop computers (19 inches, Core i5, RAM: 4GB, CPU: 3.20 GHS) to perform the computerized online interpretation task. After reading the task instructions, participants performed the training phase of the interpretation task to make sure that participants learned how to respond. The results of the training phase were not included in the analysis. Next, participants completed the main phase of the interpretation task. Upon completion of the task, participants were debriefed, and the session was terminated. After testing all subjects in the illness-anxious group, recruitment of the control group was started. The procedures for selecting participants without illness-anxiety symptoms and collecting data were the same as the procedures for the illness-anxious group.

## Results

### Data preparation for the online interpretation task

The frequency of chosen safe and unsafe word resolutions for ambiguous, health-related, and non-health-related scenarios, as well as the mean reaction time to each scenario type, was extracted using MATLAB R2017a. The frequencies of correct and incorrect (error) answers for forced health-related and non-health-related trials were calculated as well. For the calculation of RTs in the forced scenarios, trials with incorrect responses were excluded.

### Data analysis

Before the main statistical analysis, the data from one participant (in the health-anxious group) were removed due to a high number of missing data. The two groups were age-matched (the health-anxious group: M = 23.20 y.o., SD = ±2.35; and the control group: M = 23.86 y.o., SD = ± 2.57).

We first aimed to evaluate the between-group differences in self-reported measures. Therefore, we used multivariate analysis of variance (MANOVA), while AS subscales (physical, mental, and social), IU subscales (Factor 1 and Factor 2), CABAH-Cat, and reappraisal were considered as the dependent variables, the group (illness-anxious vs. control group) was considered as the fixed factor. The result of Box's M-test was not significant (*p* = 0.86), and the MANOVA assumption of homogeneity of covariance was approved. The Pillai's trace test was significant [V = 0.57, F (7, 37) = 9.59, *p* = 0.001, ${{\text{η}}_{\text{p}}}^{2}$ = 0.57], revealing that the between-group difference was meaningful at least in one variable. According to the MANOVA results, the illness-anxious group, in comparison to the control group, significantly reported higher levels of AS-physical [F (1, 56) = 52.55, *p* = 0.001, ${{\text{η}}_{\text{p}}}^{2}$ =0.48], AS-mental [F (1, 56) = 32.93, *p* = 0.001, ${{\text{η}}_{\text{p}}}^{2}$ = 0.37], AS-social [F (1, 56) = 4.60, *p* = 0.03, ${{\text{η}}_{\text{p}}}^{2}$ = 0.07], IU-factor 1 [F (1, 56) = 17.99, *p* = 0.001, ${{\text{η}}_{\text{p}}}^{2}$ = 0.24], IU-Factor 2 [F (1, 56) = 19.45, *p* = 0.001, ${{\text{η}}_{\text{p}}}^{2}$ = 0.25], and CABAH-Cat [F (1, 56) = 44.01, *p* = 0.001, ${{\text{η}}_{\text{p}}}^{2}$ = 0.44], as well as lower levels of reappraisal [F (1, 56) = 9.20, *p* = 0.004, ${{\text{η}}_{\text{p}}}^{2}$ = 0.14]. The results are presented in Table 1.

**Table 1:** Results of descriptive statistics and MANOVA for between-group differences in self-report variables: AS-physical, AS-mental, AS-social, IU-Factor 1, IU-Factor 2, reappraisal, and CABAH-Cat.

	**Group**						

	**Illness-anxious**		**Control**				
	**(*****n*** = **29)**		**(*****n*** = **29)**				
	**M**	**SD**	**M**	**SD**	**SS**	**MS**	**F**
AS-Physical	16.44	5.71	6.68	4.45	1,380.84	1,380.84	52.55^***^
AS-Mental	7.24	30.80	2.58	2.14	314.22	314.22	32.93^***^
AS-Social	9.20	2.92	7.62	2.70	36.48	36.48	4.60^*^
IU-Factor 1	48.20	12.81	36.31	8.00	2,052.15	2,052.15	17.99^***^
IU-Factor 2	40.10	9.81	30.65	6.06	1,294.41	1,294.41	19.45^***^
Reappraisal	23.51	5.01	27.55	5.11	236.01	236.01	9.20^**^
CABAH-Cat	22.68	6.66	12.75	4.53	1,430.06	14,30.06	44.01^***^

*** *p* < 0.001,

** *p* < 0.01,

** *p* < 0.05; SS, Sum of Squares; MS, Mean of Squares.

AS, Anxiety Sensitivity; IU, Intolerance of Uncertainty; CABAH-Cat, Catastrophic cognition subscale of Cognitions about body and health questionnaire.

Then, we used MANOVA to test our second hypothesis on the between-group differences in the valence of resolutions in the online interpretation task, including safe resolution for ambiguous scenarios (AMB-safe), unsafe resolution for ambiguous scenarios (AMB-unsafe), threatening resolution for health-related scenarios (Forced: HR-unsafe), and safe resolution for non-health-related scenarios (forced: NHR-safe). The valences were the dependent variables, while the group was the fixed factor. MANOVA assumption of homogeneity of covariance was approved (*p* = 0.18). The Pillai's trace test was significant [V = 0.26, F (4, 53) = 4.75, *p* = 0.002, ${{\text{η}}_{\text{p}}}^{2}$ = 0.26], revealing that the between-group difference was meaningful at least in one variable. MANOVA results are reported in Table 2. Results demonstrated that individuals with illness anxiety significantly selected less safe [F (1, 56) = 16.54, *p* = 0.001, ${{\text{η}}_{\text{p}}}^{2}$ = 0.22] and more unsafe, that is, health-threatening [F (1, 56) = 10.47, *p* = 0.002, ${{\text{η}}_{\text{p}}}^{2}$ = 0.15] resolutions for ambiguous scenarios in comparison to the control individuals. There was no significant between-group difference in the valence of resolution of forced health-related and non-health-related scenarios. Between-group differences on the error variables in the forced inference descriptions were not significant for either the safe resolution for the health-related scenario [F (1, 56) = 0.95, *p* = 0.33, ${{\text{η}}_{\text{p}}}^{2}$ = 0.01] or the unsafe resolution for the non-health-related scenario [F (1, 56) = 1.23, *p* = 0.27, ${{\text{η}}_{\text{p}}}^{2}$ = 0.2].

**Table 2:** Results of descriptive statistics and MANOVA for between-group differences in task-related variables: AMB-Safe, AMB-Unsafe, HR-Unsafe, and NHR-Safe.

	**Group**						

	**Illness-anxious**		**Control**				
	**(*****n*** = **29)**		**(*****n*** = **29)**				
	**M**	**SD**	**M**	**SD**	**SS**	**MS**	**F**
AMB-Safe	4.51	2.38	7.37	2.94	118.77	118.77	16.54^***^
AMB-Unsafe	10.37	2.55	8	3.02	82.08	82.08	10.47^**^
HR- Unsafe	7.58	0.73	7.51	0.68	0.06	0.06	0.137
NHR-Safe	7.48	0.78	7.68	0.6	0.62	0.62	1.266

*** *p* < 0.001,

** *p* < 0.01,

** *p* < 0.05; SS, Sum of Squares; MS, Mean of Squares.

AMB, ambiguous; HR, health-related; NHR, non-health-related.

To test the between-group differences in the reaction time (RT) to each scenario type, a 2 (Group) × 3 (Scenario-types-RT) MANOVA was applied while the group was specified as the between-subject factor and mean reaction times to scenario types (AMB, HR, and NHR) as the within-subject variables. Box's M (*p* = 0.98) and Mauchly's test of sphericity (*p* = 0.58) were both nonsignificant, approving the homogeneity of covariance and within-subject covariance equality, respectively. Results revealed that there was a significant effect of scenario-type-RT [F (2, 112) = 50.96, *p* = 0.001, ${{\text{η}}_{\text{p}}}^{2}$ = 0.47] and its interaction with the group [F (2, 112) = 3.61, *p* = 0.03, ${{\text{η}}_{\text{p}}}^{2}$ = 0.06]. *Post-hoc* tests demonstrated that all participants significantly reacted slower to ambiguous scenarios compared with HR and NHR scenarios (Table 3).

**Table 3:** Results of pairwise comparisons for reaction time to interpretation bias task trials for all participants (*n* = 58).

**(I) Trial**	**(J) Trial**	**Mean Difference (I-J)**	**Std. Error**	**Sig**.
AMB	HR	253.09	34.06	0.001
	NHR	284.46	25.74	0.001
HR	AMB	−253.09	34.06	0.001
	NHR	31.36	32.25	0.33
NHR	AMB	−284.46	25.74	0.001
	HR	−31.36	32.25	0.33

AMB, ambiguous; HR, health-related; NHR, non-health-related.

To identify the scenario types that the groups reacted to with different speeds, we used MANOVA after analyzing the homogeneity of covariance (*p* = 0.98) and the Pillai's trace [V = 0.13, F (3, 37) = 2.67, *p* = 0.057, ${{\text{η}}_{\text{p}}}^{2}$ = 0.13]. Scenario-types-RT was the dependent variable while the group was the fixed factor. Results showed that the illness-anxious individuals significantly had a higher reaction time (slower) to non-health-related (NHR) scenarios compared with the control participants. The two groups' reaction times to ambiguous and health-related scenarios were not significantly different. A summary of these findings is presented in Table 4.

**Table 4:** Results of descriptive statistics and MANOVA for between-group differences in Reaction Time to AMB, HR, and NHR scenarios.

	**Group**						

	**Illness-anxious**		**Control**				
	**(*****n*** = **29)**		**(*****n*** = **29)**				
	**M**	**SD**	**M**	**SD**	**SS**	**MS**	**F**
AMB-RT	1,650.93	356.93	1,541.69	299.28	173,015.67	173,015.67	1.59
HR-RT	1,349.22	337.24	1,337.20	324.09	2,097.86	2,097.86	0.01
NHR-RT	1,400.56	373.37	1,223.13	247.18	456,466.68	456,466.68	4.55^*^

* *p* < 0.05.

AMB, ambiguous; HR, health-related; NHR, non-health-related.

In the next step, we evaluated if the illness-anxious and the control group had different reaction times when they selected resolutions with safe and unsafe valences for ambiguous scenarios. One of the individuals in the illness-anxious group had no safe resolution and one of the participants in the control group had no unsafe resolution for ambiguous scenarios. Therefore, these two reaction times were considered missing data before the analysis. We did a 2 (Group) × 2 (Ambiguous-resolution-RT) MANOVA while the group was specified as the between-subject factor and reaction time to safe and unsafe resolutions of ambiguous scenarios as the within-subject variables. Box's M (*p* = 0.70) was non-significant approving homogeneity of covariance. Results demonstrated that there was a significant effect of ambiguous-resolution-RT [F (1, 54) = 9.22, *p* = 0.004, ${{\text{η}}_{\text{p}}}^{2}$ = 0.14] but not its interaction with the group [F (1, 54) = 0.49, *p* = 0.48, ${{\text{η}}_{\text{p}}}^{2}$ = 0.009]. Participants in both groups significantly reacted faster to unsafe resolutions [M = 1,559.55, SD = ± 351.33] than safe resolutions [M = 1,672.45, SD = ± 379.20]. The mean reaction time for the illness-anxious group was [Unsafe: M = 1,613.30, SD = ± 367.93; Safe: M = 1,752.32, SD = ± 407.44] and for the control group was [Unsafe: M = 1,505.80, SD = ± 331.78; Safe: M = 1,592.59, SD = ±337.16].

After revealing the between-group differences in the developmental and maintenance factors, we aimed to explore our research question about the identification of the variables that could appropriately discriminate between individuals in the illness-anxious and control groups. Therefore, a discriminant analysis was performed on self-report and IB task-related variables separately.

The discriminant analysis was first performed on the self-report variables of AS-physical, AS-mental, AS-social, IU-factor1, IU-factor2, CABAH-Cat, and reappraisal. The results of the equality of group means, assessed by the MANOVA option of discriminant analysis, were the same as in Table 1. Wilks' Lambda (0.42) was significant (*p* = 0.001), showing that the groups were different according to their means in the mentioned questionnaires. The canonical correlation (0.75) revealed that the correlation between the discriminant scores and the dependent variable was high. The Chi-square and Eigenvalues were 44.70 and 1.34, respectively, for this canonical correlation. The absolute size correlation for each variable has been presented in Table 5. These discriminant variables could correctly classify 89.7% of the subjects (*n* = 29) in the illness-anxious group and 89.7% of the subjects (*n* = 29) in the control group.

**Table 5:** Within-groups correlations between self-report discriminating variables and standardized canonical discriminant functions.

	**Function**
	**1**
AS-Physical	0.83
CABAH-ICat	0.76
AS-Mental	0.66
IU-Factor 2	0.50
IU-Factor 1	0.48
Reappraisal	−0.35
AS-Social	0.24

AS, Anxiety Sensitivity; IU, Intolerance of Uncertainty; CABAH-INT cat, Catastrophic Cognition subscale of Cognitions about body and health questionnaire.

Then, we performed the discriminant analysis for two IB task-related variables that were found to differ significantly between the two groups, that is, the numbers of safe and unsafe resolutions (valence) for ambiguous scenarios (AMB-safe and AMB-unsafe). The results of the equality of group means, assessed by the MANOVA option of discriminant analysis, were the same as in Table 2. Wilks' Lambda (0.74) was significant (*p* = 0.001), showing that the groups were different according to their means in these task-related indices. The canonical correlation (0.50) revealed that the correlation between the discriminant scores and the dependent variable was moderate. The Chi-square and Eigenvalues were 16.26 and 0.34, respectively, for this canonical correlation. The absolute size of the correlation for each variable is presented in Table 6. These discriminant variables could correctly classify 72.4% of the subjects (*n* = 29) in the illness-anxious group and 69% of the subjects (*n* = 29) in the control group.

**Table 6:** Within-groups correlations between discriminating variables and standardized canonical discriminant functions.

	**Function**
	**1**
AMB-Safe	0.92
AMB-Unsafe	−0.73
AMB: Ambiguous	

## Discussion

The current study aimed to study the role of cognitive factors in the development and maintenance of illness anxiety symptoms by going beyond mono-method assessment and using indirect measures of interpretation bias to bodily symptoms. We evaluated the contribution of developmental factors (AS and IU) and maintaining factors (interpretation bias and reappraisal) to illness anxiety disorder. The illness-anxious group reported higher levels of AS and IU and interpreted ambiguous health-related situations more catastrophically compared with the control group. Both self-report catastrophic interpretation and the online interpretation task, which is an indirect measure of biased interpretation, supported this finding. Illness-anxious participants used less reappraisal to regulate their emotions than participants in the control group. Interestingly, the illness-anxious individuals processed non-health-related situations longer than the control group, reflected in their higher reaction time to these scenarios. We also aimed to go beyond evaluating the relationship between illness and cognitive factors by assessing which factors might contribute more to the difference between people with and without illness anxiety symptoms. The results indicated that the physical subscale of AS and individuals' reaction to the safe resolution of the ambiguous scenario were among the best factors that could differentiate individuals with illness anxiety from others.

Our findings are in line with the findings and suggestions of several previous studies. Fergus and Bardeen (38) studied the incremental specificity of AS and IU with health anxiety using a large sample of adults and endorsed that both AS and IU incrementally contribute to the prediction of health anxiety. However, they demonstrated that only physical AS and inhibitory IU (corresponding to factor 1 in IUS-27) had a unique relation with health anxiety. The unique relationship between illness anxiety and AS-physical has been supported in different studies, suggesting that the role of the physical domain of AS might be more prominent in illness anxiety than the other domains (38–42). Although in our research, AS-physical had a higher correlation with discriminant function (group), all other sub-factors of AS and two factors of IU were significantly different between the illness-anxious and control groups. Our results supported the idea that AS is a fundamental fear and people with different emotional problems can experience AS as a whole construct regardless of its domains (31, 43, 44). Our results about the significant contribution of AS and IU to illness anxiety also questioned the claim raised by Fergus and Bardeen (38) that the importance of AS and IU in illness anxiety might have been overestimated.

Sensitivity to uncertain bodily sensations might be related to the vulnerability to biased processing of health-related information and an increment of anxiety over somatic sensations (45). Supporting the presence of interpretation bias in the illness-anxious individuals, our results indicated that participants with illness anxiety interpreted ambiguous health-related information as more threatening than individuals without illness anxiety. These results were observed both in the self-report and the task-related indices of interpretation bias. Although our study did not aim to study reflective and automatic processes, self-report and task-related misinterpretation in illness anxiety might support the idea that individuals with illness anxiety show reflective and automatic interpretation biases for health-related information. It is believed that the amount of effort that someone allocates to information processing distinguishes the processes of interpretation at automatic and reflective levels (46). Self-report measurements assess explicit/reflective processes, while fast-paced tasks evaluate more spontaneous and automatic interpretative processing (47). In our study, we used both a self-report questionnaire and a fast-paced online task to measure interpretation bias. As mentioned earlier, our research was not designed to evaluate reflective vs. automatic processes, and further studies are required to investigate this topic appropriately designed for.

In the online interpretation bias task, the illness-anxious individuals showed higher unsafe and lower safe interpretations of ambiguous health-related information than the control individuals. Consistent with our findings, a positive correlation between health-threatening resolution of the online ambiguous health-related information and fear of pain was reported (27). Furthermore, a systematic review by Leonidou and Panayiotou (48) supported the association between interpretation bias for health-threatening information and illness anxiety. Miles et al. (49) reported that individuals with high fear of cancer considered more negative interpretations of ambiguous cancer-related scenarios in comparison with those having lower levels of fear of cancer. However, between-group differences in Miles et al.'s study (49) were not significant for positive interpretations. The between-group difference, however, was not significant regarding the other indices of interpretation bias (i.e., faster reaction time in choosing the unsafe resolution of ambiguous scenarios than safe resolutions). Both groups reacted slower to the ambiguous scenarios when compared with the forced scenarios, while there were no between-group differences in reaction time to ambiguous scenarios. However, the study showed that the illness-anxious individuals processed NHR-forced scenarios significantly slower than the control group. It might be speculated that people with illness anxiety are inclined to look for health-related resolutions even in evident non-health-related situations and take more processing time to make sure that no threatening interpretation is possible. This finding has not been reported previously and the present results on within-subject differences provide relative support for the aforementioned explanation. Consistent with Vancleef et al.'s (27) study, our results demonstrated that both the illness-anxious and control groups reacted slower to ambiguous scenarios in comparison with HR and NHR ones.

Our results demonstrated that both groups selected unsafe resolutions faster than safe resolutions for ambiguity. Consistent with this finding, Vancleef et al. (27) reported that all participants reacted faster to the threatening resolutions of ambiguous health-related situations than the safe resolutions. An automatic threat evaluation system (TES) makes people vigilant to potentially threatening information (50). Even though our task encompassed control over non-health-related scenarios, the ambiguous and forced health-related scenarios might have acted as unintentional primes for the activation of these mechanisms and resulted in faster responses to unsafe resolutions in all participants, irrespective of the level of illness anxiety (27).

Our findings also suggest that illness-anxious individuals may have problems with reappraisal strategies to replace unsafe interpretations with safer ones. These findings are also congruent with the theories that argue the negative interpretation of health-related information is associated with reappraisal abilities (19, 20). Bardeen and Fergus (51) demonstrated that less application of reappraisal strategy was associated with higher levels of concerns about health and preoccupation with bodily sensations. Therefore, individuals with illness anxiety not only experience misinterpretation of health-related information but also lack functional reappraisal to lessen the negative emotional consequences of such misinterpretations.

Discriminant analyses showed that all the aforementioned variables could discriminate illness-anxious individuals from non-anxious individuals. AS-physical and safe resolutions for ambiguous scenarios were the best self-report and task-related discriminators, respectively, among other measures. The discriminative ability of self-report questionnaires was higher than that of task-related indices. This difference might be due to the overlap between illness anxiety symptoms and the items of the questionnaires used for the assessment of AS, IU, and self-report interpretation bias. For example, Taylor (37) argued that ASI assesses the same construct that health anxiety targets. Furthermore, the difference between self-report and task-related indices in distinguishing the individuals might be considered as the different roles of systematic and automatic cognitive processes, respectively, in illness anxiety. In other words, it might be hypothesized that individuals might attribute negative interpretations to ambiguous situations automatically regardless of their illness anxiety level. However, it seems that their levels of AS, IU, and regulatory strategies might be involved in the development of illness anxiety. This assumption is speculative and needs further investigation. Moreover, when we entered the scores of the health anxiety inventory into the analysis, the correctness of group allocation increased from 89 to 96%. It indicates that there might be other factors contributing to illness anxiety rather than AS, IU, reappraisal, and interpretation bias.

The findings of the present study should be interpreted in light of the following limitations. Participants' personal experiences of health-related issues were not controlled for. Therefore, it is not clear if their resolutions for a situation are an interpretation bias or an association of their personal experiences. Both illness-anxious and control groups were university students and caution is warranted in generalizing the findings to the clinical and general population. Furthermore, the sample was recruited through a volunteer catchment at the university rather than selecting more diverse clients who are seeking help in clinics. Therefore, the level of dysfunctionality reported by our sample might be less than the clinical population. This may increase the chance of type-II error in our analyses, which has to be considered when we interpret the results. The other limitation of the current study was the small sample size. Regarding the number of statistical comparisons, having a bigger sample size would reduce the chance of type-II error. Item overlap across some of our measures, such as ASI and CABAH, might be another statistical issue. However, CABAH items mostly measure “beliefs” about somatic sensations and health while ASI evaluates the “fear” of these sensations. In addition, regarding the number of comparisons we included in our study, having a larger sample size would be more beneficial in estimating more precise effect sizes.

Despite these limitations, the current study is one of the few works that used multi-method assessment to highlight the role of different developmental and maintenance constructs in the psychopathology of illness anxiety. Considering that direct measures of interpretation bias in previous studies were prone to response bias, we evaluated the interpretation bias as an indirect process using an online reading task. The study investigated if interpretation bias, reappraisal, AS, and IU could differentiate the illness-anxious and control individuals. Based on the results, AS and IU might moderate the attention to and perception of ambiguous bodily sensations or somatosensory amplification. The efforts of the individual to resolve this ambiguity, in turn, may prone the person to more negative interpretation bias for this ambiguous sensation and increase anxiety. Deficits in the reappraisal strategy of emotion regulation, then, prevent the individual from replacing these unsafe interpretations with safe ones to reduce anxiety levels. They might try to gain certainty or decrease the levels of anxiety by dysfunctional strategies such as safety-seeking behaviors or avoidance that will lead to illness anxiety disorder in the long term. The methods and findings of the current study can be addressed in future studies for a more comprehensive understanding of illness anxiety and the application of its treatment. For instance, in the current study, there were no between-group differences in reaction time to ambiguous situations. Further studies can investigate if the reaction time might be different in the clinical sample who probably experience the symptoms more severely. Our results showed that people with illness anxiety symptoms are generally interpreting ambiguous situations in negative ways. However, it can be examined if illness-anxious individuals may interpret some ambiguous health-related situations as more catastrophic than other ambiguous health-related situations. The results will reveal the within-individual differences in catastrophic interpretations of ambiguous health-related situations, leading to identifying more specific factors that are involved in illness anxiety. Regarding the role of interpretation bias in illness anxiety, cognitive behavioral therapy (CBT) protocols target these biases by asking patients to evaluate the meaning of bodily sensations and adopt less-threatening interpretations (52). Considering that CBT strategies require higher levels of cognitive resources for introspection and awareness, they may not be as effective as more automatic and habitual level strategies in more stressful situations with high cognitive loads. Accordingly, there is a need for developing indirect and online techniques that address the interpretation biases more subtly and without requiring effortful introspection.

## Conclusion

The current study highlighted the role of cognition in the development and maintenance of illness anxiety symptoms. Our results revealed that the illness-anxious group associated ambiguous health-related situations with more catastrophic consequences than the control group. Compared with the control group, the illness-anxious individuals also tended to be more sensitive to physical and cognitive symptoms of anxiety and showed less tolerance to general ambiguous situations. Sensitivity to the physical symptoms of anxiety and less safe interpretations for ambiguous health-related situations were among the best factors that could differentiate individuals with illness anxiety from others. These findings provide important suggestions for future interventions aiming to reduce anxiety and improve the quality of life among those patients suffering from conditions that raise the possibility of health anxiety. Changing the health-threatening interpretation of ambiguous bodily symptoms to more neutral interpretations might be a possible way to reduce health-related anxiety and improve patients' quality of life.

## Data availability statement

The raw data supporting the conclusions of this article will be made available by the authors, without undue reservation.

## Ethics statement

The studies involving human participants were reviewed and approved by Department of Psychology, Shahid Behesht University. The patients/participants provided their written informed consent to participate in this study.

## Author contributions

ME was involved in the design, conducting study, analysis, and writing. MD was involved in design, supervision, and writing. MH was involved in design, analysis, and writing. LV was involved in material development and writing. AK was involved in design, material development, supervision, analysis, and writing. All authors contributed to the article and approved the submitted version.
