# Supplementary material for: Negative interpretation of ambiguous bodily symptoms among illness-anxious individuals: Exploring the role of developmental and maintenance constructs
Source: Front Psychiatry. 2023 Jan 9;13:985125. doi: 10.3389/fpsyt.2022.985125 (PMC9868299; doi:10.3389/fpsyt.2022.985125)
Supplement: Supplementary file 1 [file Table_1.DOCX]

**Appendix A**

Below, we have presented examples of four-line ambiguous, health-related, and non-health-related scenarios that were used in the Interpretation task. The main task was in Farsi. Here, we have presented the word-to-word English translation of Farsi scenarios. Due to linguistic and cultural differences, the English translations might not make sense in some respects.

|  | Ambiguous scenario | Health-related Control scenario |
| --- | --- | --- |
| 1 | There are many people at the gathering.  Your best friend’s mother and father  are crying.  Your best friend ---.   1. Has died 2. Has married | You have read a very good novel.  You would like to talk to its author  but unfortunately, you cannot  because two years ago, the author ---.   1. has died 2. has married |
| 2 | The man's forehead is sweaty.  His heart is pounding,  and He is breathing deeply.  This is the result of ---.   1. heart attack 2. Running | Your dad walks regularly  and eats in a balanced way.  A healthy lifestyle at this age  is necessary to avoid ---.   1. heart attack 2. Running |
| 3 | Sina continues to cough heavily.  He has had this problem for a while.  This condition is sometimes accompanied by pain.  Cough is caused by ---.   1. smoking 2. Lung cancer | Smoking is bad for your health.  It can lead to a variety of lung problems.  In the worst case,  it can lead to ---.   1. smoking 2. Lung cancer |
| 4 | The film actor is nauseous  and vomits frequently.  Also, his reaction to the words of the other actor is slow.  He has been ---.   1. Poisoned 2. drunk | On the way back from university, you felt hungry.  Therefore, you went to a fast food.  But after eating, you feel nauseous.  It looks like you are ---.   1. Poisoned 2. drunk |
| 5 | You see a woman on the street.  It takes a lot of effort for her  to cross the curb.  it is difficult to cross the curb with ---.   1. Wheelchair 2. Baby stroller | There is a tennis competition for disabled people.  You are watching the game.  These two players  have been sitting all their lives on ---.   1. Wheelchair 2. Baby stroller |
|  | Ambiguous scenario | Non_Health-related Control scenario |
| 6 | You fell while you were walking.  When you stood up,  you felt as if your knee is wet.  Your knee was ---.   1. muddy 2. bloody | It rained all day.  You went for a walk in the woods.  When you return,  your shoes were ---.   1. muddy 2. bloody |
| 7 | You are talking to a skier.  He competed in various international competitions.  In one of the matches,  he broke his ---.   1. Record 2. leg | An athlete went to the Olympics.  He won after a beautiful performance.  In addition, once again,  he broke his ---.   1. Record 2. leg |
| 6 | You saw your old friend after a long time.  Surprisingly, he is completely bald.  He told you that  his baldness is because of ---.   1. Chemotherapy 2. Acting makeup | Your brother is an actor.  A few days ago, an experienced makeup artist,  made him up.  He is satisfied with his ---.   1. Chemotherapy 2. Acting makeup |
| 8 | A woman is coming toward you  and she is trying  to keep his balance.  Walking with --- is very difficult.   1. Cane 2. High heels | One of your friends is 180 cm tall.  She considers herself very tall.  For this reason,  she never wears ---.   1. Cane 2. High heels |
| 10 | Yesterday, you collided with a car while riding a bicycle. You cannot now ride a bike.  In that accident,  you broke your ---.   1. Bike 2. leg | The weather is good  and the sun is shining.  This good weather  Is suitable for ---.   1. (riding) Bike 2. leg |
